# COM33 suppresses carboplatin-induced epithelial-mesenchymal transition via inhibition of Twist1 in ovarian cancer: COM33 sensitizes ovarian cancer to carboplatin

**DOI:** 10.3724/abbs.2022195

**Published:** 2022-12-22

**Authors:** Zhiyang Zhou, Li Jin, Jian Shen, Weihui Shi, Yue Xu, Longyun Ye, Junxi Liu, Jiexue Pan

**Affiliations:** 1 Obstetrics & Gynecology Hospital Institute of Reproduction and Development Fudan University Shanghai 200011 China; 2 College of Life Science Zhejiang Chinese Medical University Hangzhou 310053 China; 3 Department of Pancreatic Surgery Fudan University Shanghai Cancer Center Shanghai 200032 China; 4 Department of Oncology Shanghai Medical College Fudan University Shanghai 200032 China; 5 Chinese Academy of Science Key Laboratory of Chemistry of Northwestern Plant Resources and Key Laboratory for Natural Medicine of Gansu Province Lanzhou Institute of Chemical Physics Chinese Academy of Sciences Lanzhou 730000 China

**Keywords:** COM33, carboplatin, ovarian cancer, epithelial-mesenchymal transition, Twist1

## Abstract

Despite favorable responses to platinum-based chemotherapy in ovarian cancer (OC), chemoresistance is still a major cause of treatment failure. Hence, we develop a novel synthetic agent, COM33, to relieve the chemoresistance caused by carboplatin. The anti-cancerous effects of the combination of COM33 and carboplatin on OC are evaluated by cell viability, wound healing, and transwell invasion assays. A mechanistic investigation is carried out by using RNA-Seq analysis and then verified by western blot analysis and immunofluorescence microscopy. The safety and efficacy *in vivo* are evaluated using SKOV3 tumor-bearing nude mice. Results show that the co-administration of COM33 enhances the inhibitory effects of carboplatin on cancer cell viability, migration, and invasion *in vitro* and tumor growth *in vivo*. Furthermore, COM33 suppresses the carboplatin-induced epithelial-mesenchymal transition (EMT) by inhibiting the ERK signaling pathway. Additionally, we show that Twist1, the effector of the ERK signaling pathway, participates in carboplatin-induced EMT and is also inhibited by COM33. Our data show that the combination of carboplatin with COM33 is beneficial for chemotherapy against OC, which may be a potential novel anti-tumor strategy.

## Introduction

Ovarian cancer (OC) is one of the most common gynecological malignant tumors and has the highest mortality rate among gynecological cancers [1]. Due to its vague clinical symptoms, OC patients are usually diagnosed in advanced stages and have poor prognoses [2]. Currently, the recommended treatment for OC is surgery combined with carboplatin/paclitaxel-based chemotherapy [3]. However, although some individuals can be cured, most patients will eventually have recurrence with distant metastasis [4]. A major reason for disease recurrence is that tumors inevitably become resistant to the chemotherapy drugs that are currently used [5]. Hence, there is an urgent need for novel agents that can sensitize chemoresistant tumors.

Furthermore, it has been reported that chemoresistance is closely associated with the epithelial-mesenchymal transition (EMT) [6]. Some researchers have proposed that chemotherapy exacerbates OC cell migration and cancer stem cell-like characteristics such as the EMT [7]. Previous studies also demonstrated that carboplatin could promote the EMT in ovarian cancer cells, enhancing the capacity for migration and invasion [ 8, 9] . EMT is defined as the loss of polarity and adhesion in epithelial cells and their shift toward the mesenchymal state, and it is widely implicated in cancer initiation, progression, metastasis, and refractory responses to chemotherapy [ 10– 13] . Moreover, Twist1 has been proposed as one of the driving factors of the EMT [14], since its overexpression downregulates E-cadherin and induces the EMT [15]. Therefore, reversing the expressions of EMT-inducing transcription factors and the carboplatin-induced EMT might be an effective way to restore the sensitivity of tumors to carboplatin.

Isocorydine (ICD) is a natural aporphine alkaloid extracted from medicinal plants. Additionally, ICD and its derivative, 8-amino-isocorydine (NICD), have been approved as antispasmodic analgesic agents which have many pharmacological effects, including anti-tumor effects [ 8, 16, 17] . In our previous study, we synthesized a novel NICD derivative, COM33, which presented anti-proliferative activity against several cancer cell lines [18]. In hepatocellular carcinoma cells, COM33 targeted cancer stem cells and the EMT process *in vivo* and *in vitro* to exert its anti-tumor activity. Additionally, COM33 could be used in combination with sorafenib to produce a synergistic effect. Thus, we postulated that COM33 might also inhibit chemoresistance in OC cells when combined with carboplatin.

In the present study, we analyzed the potential benefits of COM33 for the treatment of OC. When combined with the conventional chemotherapy drug carboplatin, COM33 presented synergistic effects by reversing carboplatin-induced EMT, thereby increasing carboplatin sensitivity in OC cells.

## Materials and Methods

### Cell lines and reagents

Human OC cell lines A1847 and SKOV3 were purchased from the Cell Bank of Shanghai Institutes for Biological Sciences (Shanghai, China). Cells were cultured in RPMI 1640 supplemented with 10% (v/v) fetal bovine serum (FBS; Gibco, Carlsbad, USA) and 1% (v/v) penicillin-streptomycin (Gibco), and were maintained in a humidified cell incubator at 37°C with 5% CO _2_.

Carboplatin (C2538) was purchased from Sigma-Aldrich (St Louis, USA) and COM33 was synthesized as previously described [18]. Antibodies against E-cadherin (4A2), Vimentin (D21H3), β-catenin (D10A8), Snail (C15D3), Claudin-1 (D5H1D), p-ERK (D13.14.4E), ERK (137F5), Twist1 (E7E2G), and GAPDH (D16H11) were obtained from Cell Signaling Technology (Beverly, USA). Antibody against Ki67 (27309-1-AP) was obtained from Proteintech (Rosemont, USA). The TUNEL assay kit was purchased from Roche (Basel, Switzerland).

Small interfering RNAs (siRNAs) were specifically designed and synthesized by RiboBio (Guangzhou, China). The sequences for *ERK* siRNA were: sense 5′-CAGGGAAGCAUUAUCUUGATT-3′ and antisense 5′-UCAAGAUAAUGCUUCCCUGTT-3′; and the sequences for *Twist1* siRNA were: sense 5′-GCAAGAUUCAGACCCUCAATT-3′ and antisense 5′-UUGAGGGUCUGAAUCUUGCTT-3′. si-NC (siN0000001-1-10) was purchased from RiboBio. The expression vector for *Twist1* was purchased from GenePharma (Shanghai, China).

### Cell viability assay

First, exponentially growing A1847 and SKOV3 tumor cells were seeded in 96-well plates at a density of 5×10 ^3^ per well and exposed to different concentrations of the compounds or the solvent (control) for 48 h. Then, Cell Counting Kit-8 (Dojindo, Japan) was added and plates were incubated for 4 h at 37°C. The optical density (OD) was read at 450 and 630 nm with a microplate reader. All experiments were conducted in triplicates. The half-maximal inhibitory concentration (IC _50_) was determined using GraphPad Prism 8.0 (GraphPad, San Diego, USA).

The Chou and Talalay method was used to calculate the combination index (CI) to determine the drug interaction degree. The formula was: CI=(D) _1_/(D _x_) _1_+(D) _2_/(D _x_) _2_. The detail was the sum of the dose of each drug when used in combination divided by the dose of each drug when used alone when the drug inhibits 50% of cell viability. A CI<1, =1, and >1 indicates synergism, additive effect, and antagonism, respectively [19].

### Wound healing assay

A1847 and SKOV3 cells were seeded in a 6-well plate. When cells were confluent, the medium was replaced by a culture medium supplemented with 1% FBS and indicated drugs. A straight wound was scratched with a 200-μL pipette tip on the confluent cell monolayer and the first image was captured. After 24 h, migrating cells were monitored under a light microscope and the final scratch distance was calculated by Image J software as an average of three different fields.

### Transwell invasion assay

Inserts with 8-μm membranes were coated with Matrigel (BD Biosciences, Bedford, USA) at 37°C for 4 h. The upper chamber with a 1% FBS medium was seeded with an equal number of treated cells, and the lower chamber was filled with a medium containing 20% FBS as a chemokine. After 24 h of incubation, the chambers containing invaded cells were fixed with 95% ethanol, washed with PBS, and stained with crystal violet. Finally, the number of cells was counted under a microscope (Olympus, Tokyo, Japan).

### RNA-Seq analysis

SKOV3 cells were treated with carboplatin or a combination of carboplatin and COM33 for 48 h. Then, total RNA was extracted with Trizol (Invitrogen, Carlsbad, USA) and used for library construction. Next, libraries were sequenced after cluster generation on an Illumina HiSeq 4000 platform (Illumina, San Diego, USA) by Shanghai Life Genes Biotech (Shanghai, China), and 150 bp paired-end reads were generated. After processing, filtering, and quality checking, differentially expressed genes (DEGs) were screened using the following criteria: *P*<0.05 and |log2 (fold change)|>1.0. Finally, differentially expressed EMT-related genes were screened for further analysis.

### Western blot analysis

Whole cells were homogenized in protein lysis buffer (P0013; Beyotime Biotechnology, Shanghai, China) after treatment with indicated compounds, and protein concentrations in the lysates were measured using a BCA Protein Assay kit (P0010; Beyotime Biotechnology). An equal amount of protein was fractionated by SDS-PAGE, and then transferred onto a PVDF membrane (Millipore, Billerica, USA). After being blocked with 5% fat-free milk for 1 h, membranes were incubated with primary antibodies at 4°C overnight. After washing, the membranes were incubated with horseradish peroxidase-conjugated secondary antibodies (Huabio, Hangzhou, China) for 1 h at room temperature and detected using ECL reagent (34095; Thermo Fisher Scientific, Waltham, USA).

### Immunofluorescence microscopy

Treated cells were cultured in a chamber slide (Millipore), and then fixed with 4% paraformaldehyde for 15 min. Next, cells were permeabilized with 0.3% Triton X-100 for 15 min, followed by blocking with 5% BSA for 30 min. Subsequently, cells were incubated with primary antibodies overnight at 4°C. After washing, slides were incubated with FITC-conjugated secondary antibody (Abcam, Waltham, USA) for 60 min at 37°C. Finally, the nuclei were stained using DAPI. Stained slides were observed under a confocal microscope (Carl Zeiss, Oberkochen, Germany).

### Xenograft experiments

All animal experiments were approved by the Institutional Animal Care and Use Committee of the Fudan University Shanghai Cancer Center. Female nude mice (3–4 weeks, BALB/c *nu*/ *nu*; Shanghai SLAC Laboratory Animal Co., Ltd, Shanghai, China) were subcutaneously transplanted with 1×10 ^6^ SKOV3 cells into the right axillary fossa. Mice were randomly divided into four groups: control, COM33 (25 mg/kg), carboplatin (40 mg/kg), and COM33+carboplatin. When the tumor was visible, drugs were intraperitoneally injected every two days. The body weight and tumor volume of each mouse were monitored every two days. Tumor volumes were measured using the following formula: V (mm ^3^)= A×B ^2^/2, where A represents the largest diameter, and B the perpendicular diameter. After two weeks of treatment, all mice were sacrificed and tumors were harvested for subsequent analyses.

### Immunochemistry staining

Tumors were fixed and embedded in paraffin. After deparaffinization, samples were rehydrated and incubated in 3% H _2_O _2_ for 15 min at room temperature. Next, tissue sections were blocked by 5% bovine serum albumin (BSA) for 30 min at 37°C. Subsequently, the sections were incubated overnight at 4°C with primary antibodies. After incubation with HRP-conjugated secondary antibody and visualization with DAB substrate, the slides were photographed at least five randomly selected fields under high magnification. The apoptosis in sections was determined by an *in situ* cell death detection kit (TUNEL; Roche) according to the standard instructions. The apoptotic cells were counted under a light microscope at five randomly selected fields.

### Statistical analysis

Data were presented as the mean±SD. The statistical significance of differences between groups was obtained by the Student’s *t*-test or ANOVA (multiple comparisons) in GraphPad Prism 8.0. All tests were two-sided and a value of *P*<0.05 was considered statistically significant.

## Results

### COM33 improves the sensitivity of OC cells to carboplatin

**Figure 1:**
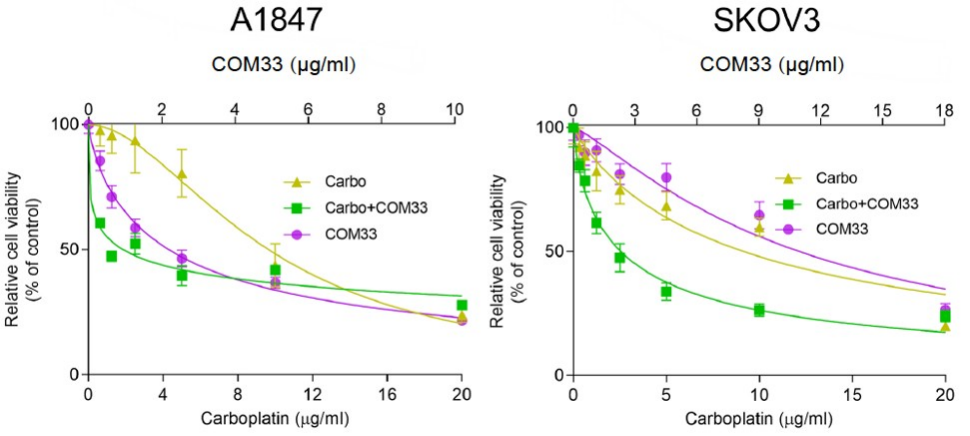
Figure 1 Anti-proliferative activities of COM33, carboplatin, and COM33/carboplatin combination against human ovarian cancer cells *in vitro* Carbo: carboplatin.

First, we tested the anti-proliferative activities of different concentrations of COM33 in A1847 and SKOV3 human OC cell lines. The cell viability was decreased with the increasing drug concentrations ( Figure 1). Then, we evaluated the effects of combining COM33 with carboplatin. The results showed that the combined treatment had a more effective anti-proliferative activity compared with COM33 or carboplatin alone ( Figure 1). The IC _50_ and combination index (CI) were summarized in Table 1. The CI was 0.625 and 0.488 for A1847 and SKOV3 cells, respectively, indicating that the addition of COM33 sensitized OC cells to carboplatin.

**Table:** **Table 1** IC _50_ values and combination index for COM33 and carboplatin in A1847 and SKOV3 cells

Cell line	IC _50_ of COM33 (μg/mL) ^a^		IC _50_ of carboplatin (μg/mL) ^b^		Combination index (CI)
	COM33	carboplatin+COM33		carboplatin	carboplatin+COM33		
A1847	1.941	0.8402**		9.658	1.867***		0.625
SKOV3	10.96	2.308**		9.193	2.564***		0.488

^a^IC _50_ of COM33 concentration in different treatments for 48 h. ^b^IC _50_ of carboplatin concentration in different treatments for 48 h. IC _50_: half maximal inhibitory. ** *P*<0.01, *** *P*<0.001.

### COM33 and carboplatin synergistically inhibit the migration and invasion of OC cells

**Figure 2:**
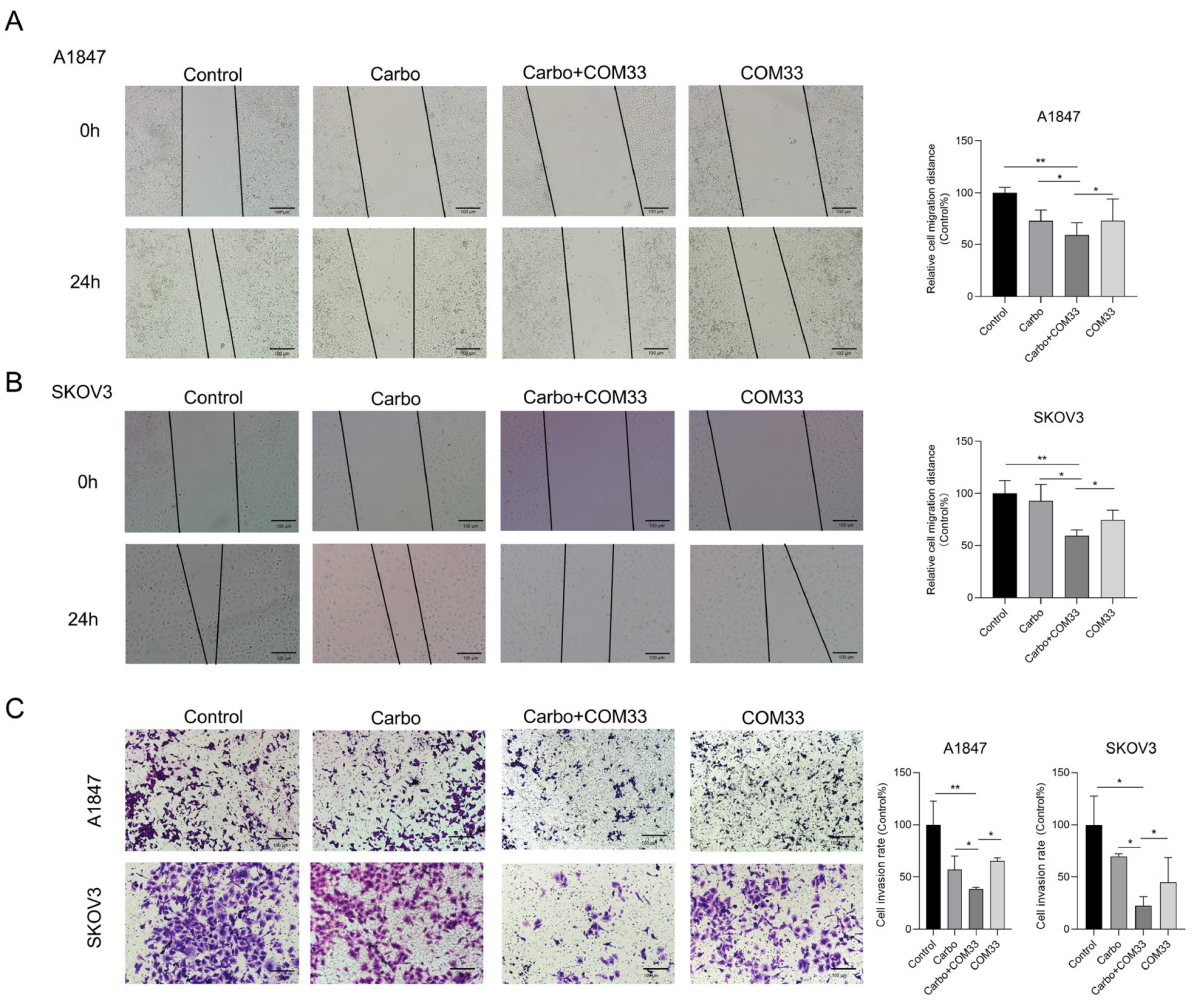
Figure 2 Anti-migration and invasion activities of COM33 and COM33/carboplatin combination against human ovarian cancer cells *in vitro* (A) Wound healing assay for A1847 cells in the control, carbo, carbo+COM33, and COM33 groups (scale bar: 100 μm). (B) Wound healing assay for SKOV3 cells in the control, carbo, carbo+COM33, and COM33 groups (scale bar: 100 μm). (C) Transwell assay for A1847 and SKOV3 cells in the control, carbo, carbo+COM33, and COM33 groups (scale bar: 100 μm). Carbo: carboplatin. * P<0.05, ** P<0.01.

Wound healing and transwell invasion assays were performed to evaluate the migration and invasive capability of cells after treatment with COM33 and carboplatin. The wound healing assays showed that COM33 plus carboplatin significantly decreased cell migration compared to COM33 or carboplatin alone ( Figure 2A,B). The transwell invasion assays also showed that the combination of COM33 and carboplatin led to a remarkably weakened invasion ability of cancer cells compared with single drugs or untreated cells ( Figure 2C).

### COM33 impairs the carboplatin-induced EMT by inhibiting ERK signaling

**Figure 3:**
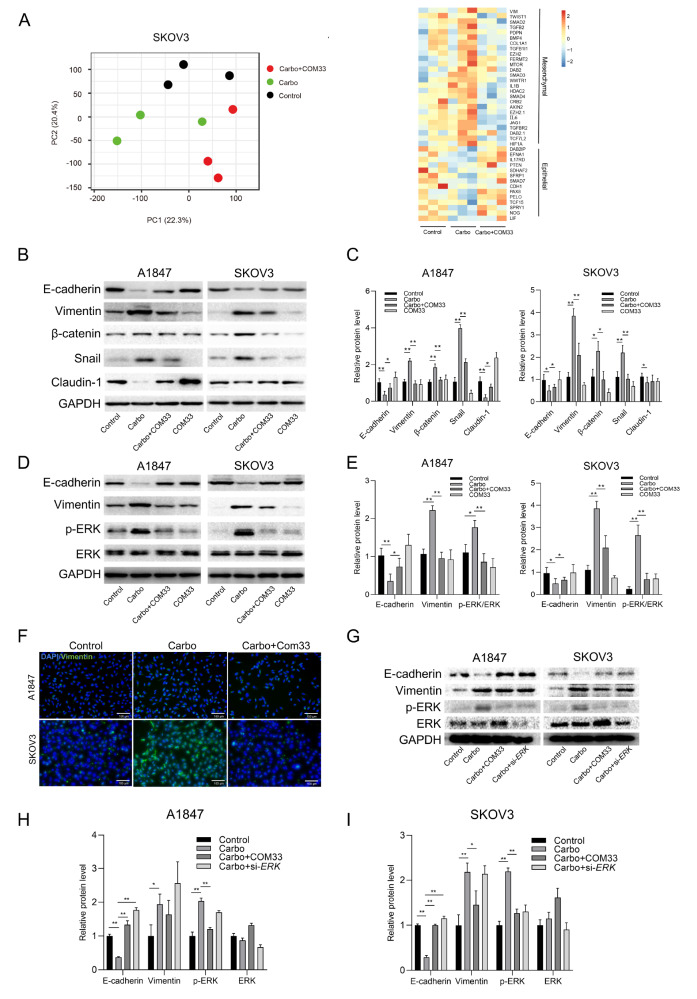
Figure 3 COM33 reverses the levels of carboplatin-induced EMT markers and ERK signaling in A1847 and SKOV3 cells (A) PCA and heat map of EMT-related genes of control, carbo, and carbo+COM33 groups in SKOV3 cells. (B,C) Detection of EMT markers in A1847 and SKOV3 cells of control, carbo, carbo+COM33, and COM33 groups by western blot analysis. (D,E) Detection of ERK signaling in A1847 and SKOV3 cells of control, carbo, carbo+COM33, and COM33 groups by western blot analysis. (F) Immunofluorescence staining of Vimentin (green) in A1847 and SKOV3 cells of control, carboplatin, and carbo+COM33 groups. The nucleus was stained with DAPI (blue) (scale bar: 100 μm). (G–I) Detection of EMT markers in A1847 and SKOV3 cells of control, carbo, carbo+COM33, and carbo+si- ERK groups by western blot analysis. Carbo: carboplatin; si- ERK: siRNA- ERK. * P<0.05, ** P<0.01.

To further elucidate the underlying mechanisms of COM33 in increasing carboplatin sensitivity, we performed RNA-seq analysis using SKOV3 cells. The principal component analysis (PCA) of the transcriptional profile showed a clear separation between the control, carboplatin, and carboplatin+COM33 groups ( Figure 3A). Next, EMT-related genes were screened out according to the Gene Ontology terms (GO: 0010718 and GO: 0010719), and the data showed that carboplatin promoted the EMT, which was reversed by COM33 addition ( Figure 3A). Then, we checked the protein expressions of EMT-related genes. After 48 h of treatment with carboplatin, both A1847 and SKOV3 cells presented significant increase of the levels of Vimentin, β-catenin, and Snail, while E-cadherin and Claudin-1 were downregulated ( Figure 3B,C). The immunofluorescence staining further confirmed the high level of Vimentin in the cytoplasm after carboplatin treatment ( Figure 3F). However, after combined treatment with carboplatin and COM33, carboplatin-induced EMT was partially reversed as evidenced by decreased levels of Vimentin, β-catenin, and Snail, and increased levels of E-cadherin and Claudin-1 ( Figure 3B,C,F), compared with those in the group received carboplatin only.

Furthermore, we analyzed the involvement of the MAPK pathway in the carboplatin-induced EMT. We found that the carboplatin-induced EMT in OC cell lines was accompanied by increased phosphorylation of ERK ( Figure 3D,E). However, the carboplatin-induced EMT was restored after *ERK* knockdown by siRNA ( Figure 3G–I). Meanwhile, when carboplatin and COM33 were combined, the carboplatin-induced EMT was reversed and the level of phospho-ERK was decreased, indicating that COM33 alleviated carboplatin-induced EMT in OC cells probably via the ERK signaling ( Figure 3G–I).

### Carboplatin-induced EMT is mediated by the transcription factor Twist1

**Figure 4:**
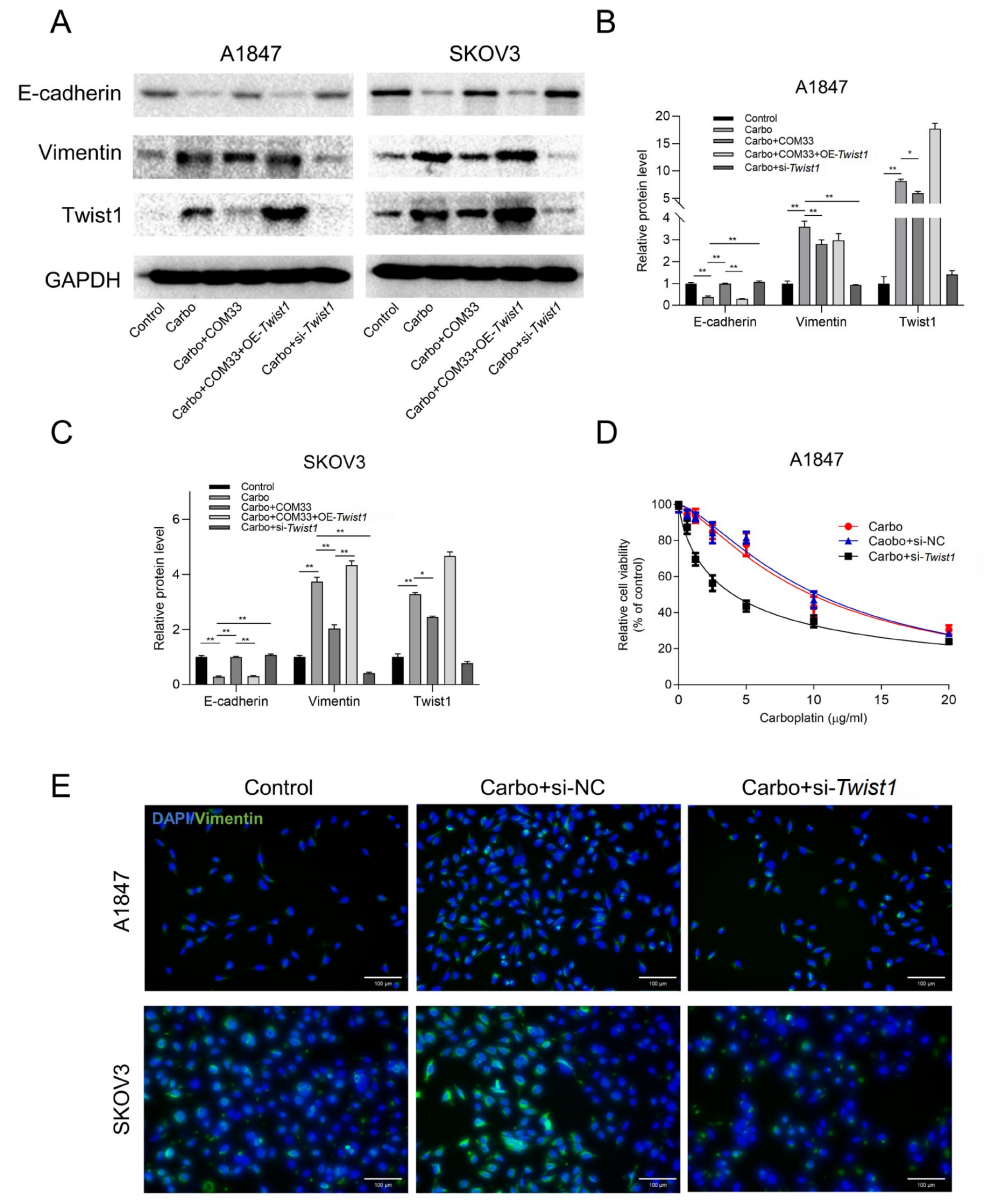
Figure 4 *Twist1* knockdown reverses carboplatin-induced EMT (A–C) Western blot analysis of Twist1 and EMT markers in A1847 and SKOV3 cells of control, carbo, carbo+COM33, carbo+COM33+overexpression of Twist1, and carbo+siRNA- Twist1. (D) Relative cell viability in groups of carbo, carbo+si-NC, and carbo+si- TWIST1 in A1847 cells. (E) Immunofluorescence staining of Vimentin (green) of control, carbo+si-NC, and carbo+siRNA- Twist1 in A1847 and SKOV3 cells. The nucleus was stained with DAPI (blue) (scale bar: 100 μm). Carbo: carboplatin; si-NC: siRNA-control; si- Twist1: siRNA- Twist1; OE- Twist1: over-expression of Twist1. * P<0.05, ** P<0.01.

The transcription factor Twist1 is an ERK signaling effector and has been demonstrated to activate the EMT, thereby driving cells toward malignancy [ 20, 21] . Here, Twist1 was upregulated in cells treated with carboplatin and the addition of COM33 suppressed this phenomenon ( Figure 4A–C). When *Twist1* was overexpressed, the effect of COM33 in inhibiting carboplatin-induced EMT was reduced, as demonstrated by the lower expressions of epithelial markers such as E-cadherin, and higher expressions of mesenchymal markers, such as Vimentin ( Figure 4A–C). On the other hand, after knockdown of *Twist1* using siRNA, the carboplatin-induced EMT was partially restored, as demonstrated by E-cadherin upregulation, and Vimentin downregulation ( Figure 4A–C,E). Subsequently, the cell viability assays showed that the resistance of A1847 cells to carboplatin was significantly reduced after knockdown of *Twist1* ( Figure 4D). Altogether, these results indicated that the carboplatin-induced EMT might be mediated by Twist1 and was inhibited by COM33.

### COM33 enhances the effects of carboplatin *in vivo*

**Figure 5:**
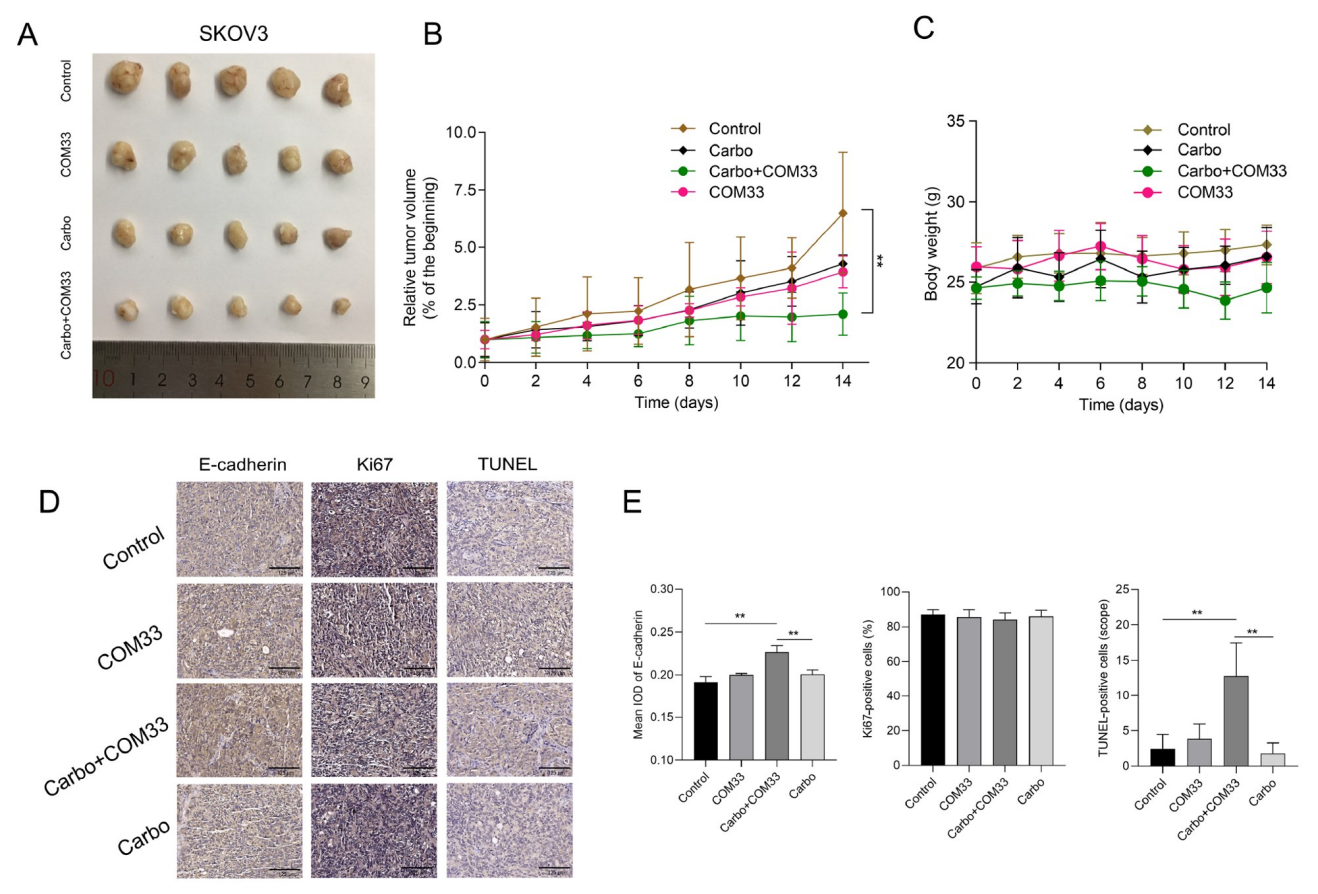
Figure 5 Effects of COM33, carbo, and their combination on SKOV3-bearing nude mice (A) Representative images of tumors in different groups. (B) Relative tumor volume ratios (% of the beginning) over time in the control group (brown), groups treated with Carbo (black), Carbo+COM33 (green), or COM33 (pink). (C) Body weight of mice after drug treatment in each group. (D) Immunohistochemical staining of E-cadherin, Ki67, and TUNEL in resultant tumors from the xenograft study (scale bar: 125 μm). (E) Immunochemistry quantifications of (D). Carbo: carboplatin; IOD: Integrated Optical Density. ** P<0.01.

To verify the role of COM33 in tumorigenesis *in vivo*, a xenograft mouse model was established using SKOV3 cells in nude mice. Once the subcutaneously implanted tumors were visible, mice were treated with vehicle, COM33, carboplatin, or COM33 plus carboplatin by intraperitoneal injection every two days and the body weight and tumor size were monitored. After two weeks of treatment, tumors were harvested from mice and photographed ( Figure 5A). The data showed that COM33 or carboplatin alone delayed tumor growth but did not reach statistical significance ( Figure 5A,B). On the other hand, the combination of COM33 with carboplatin inhibited the growth of xenografts as indicated by the final tumor volume and the tumor growth curves ( Figure 5A,B). We also found that the body weight of mice was slightly changed after drug treatment ( Figure 5C). Overall, the combination of COM33 with carboplatin presented promising anti-tumorigenesis abilities *in vivo*.

The subsequent xenograft tumor analyses showed that the expression of E-cadherin was significantly increased after combining carboplatin with COM33 compared with the control and carboplatin-treated groups ( Figure 5D,E). Although the fraction of proliferating cells labeled Ki67 was similar among groups, the TUNEL staining revealed a marked increase in apoptotic cells after administration of carboplatin plus COM33 compared with control or carboplatin-treated groups ( Figure 5D,E).

## Discussion

The development of high-throughput sequencing technologies provides a rapid and economical way to identify tumor genomic profiling. Targeted therapies based on molecular tumor profiling are now integrated into treatment guidelines for many solid tumors including OC [22]. For example, PARP inhibitors have been approved as first-line maintenance therapy, especially among patients who harbor a germline or somatic *BRCA* mutation [23]. Nevertheless, a greater understanding of the molecular landscape of OC and developing robust clinical trials to assess the effectiveness of emerging targeted drugs requires further research [24].

Although targeted therapies provide alternatives that improve efficacy and minimize toxicity, platinum-based chemotherapy remains the standard treatment for OC due to its sensitivity. Unfortunately, drug resistance has become the main reason for failures. Some researchers have revealed several molecular mechanisms of platinum-based drug resistance, including the elimination of intracellular toxic drugs, activation of DNA repair, and mutation of certain oncogenes [ 25– 27] . Previous studies have demonstrated that carboplatin could promote EMT [ 8, 9] . This observation was also confirmed in the present study. These results demonstrated that the EMT might be one of the mechanisms by which OC cells become resistant to carboplatin.

To overcome acquired drug resistance, researchers are focusing on developing new agents and their combinations, aiming to finally reduce clinical mortality. COM33 is a novel compound synthesized via structural modifications of NICD [18]. These modifications improve the characteristics of NICD (easily oxidized in water due to its p-aminophenol substructure) to better exert its pharmacological effects. In the present study, we demonstrated that COM33 decreased the viability of OC cells in a dose-dependent manner, but had no obvious effect on migration and invasion at a low dose ( Figures 1 and 2). In addition, in the xenograft experiments, we found that, although the low-dose COM33 alone delayed tumor growth, it did not statistically differ from the control group ( Figure 5A,B). Next, we verified whether COM33 could play a synergistic anti-tumor role with carboplatin, and explored the related mechanisms. In the *in vitro* experiments, we found that COM33 and carboplatin could synergistically inhibit the viability of tumor cells with CI of 0.625 and 0.488 for A1847 and SKOV3 cells, respectively ( Table 1). At the same time, their combined use could significantly inhibit the migration and invasion of OC cells ( Figure 2). In the *in vivo* experiments, we found that COM33 plus carboplatin inhibited the growth of xenografts compared with the control group ( Figure 5). These results indicated that the co-administration of COM33 with carboplatin was effective. Finally, we found that COM33 could reverse the EMT and inhibit the ERK signaling pathway that was induced by carboplatin ( Figure 3D,E), indicating that COM33 could reverse carboplatin resistance. This could partially explain why COM33 and carboplatin had a combinational effect.

Furthermore, Twist1 is a basic helix-loop-helix (bHLH) domain-containing transcription factor that has been proven to be an ERK effector and one of the master regulators of the EMT [ 20, 28] . The link between Twist1 and enhanced OC metastasis has also been established [29]. Here, we showed that the carboplatin-induced EMT might be mediated by Twist1 and was inhibited by COM33.

In summary, we demonstrated that COM33 could inhibit the chemoresistance to carboplatin by reversing carboplatin-induced EMT and reducing OC cell proliferation, migration, and invasion. Our results provided evidence that COM33 is a promising combinational drug to treat OC.
